# Happiness in the Homeland: Satisfaction with Life And Its Correlation with Flourishing and Affect Balance in Foreign-Trained, Repatriated Physicians in Pakistan

**DOI:** 10.7759/cureus.101043

**Published:** 2026-01-07

**Authors:** Madah Fatima, Ali Madeeh Hashmi, Muhammad Talha Farooq, Aiman Javed

**Affiliations:** 1 Academic Department of Psychiatry and Behavioral Sciences, Mayo Hospital, King Edward Medical University, Lahore, PAK; 2 Psychiatry, Gartnavel Royal Hospital, Glasgow, GBR

**Keywords:** affect balance, flourishing, international medical graduates (imgs), repatriated physicians, satisfaction with life

## Abstract

Introduction

International medical graduates (IMGs) constitute a significant proportion of the global physician workforce, with Pakistan as a leading source country. Despite many Pakistani physicians emigrating for training and employment, limited research exists on the well-being of those who return. This study aimed to assess satisfaction with life (SWL) in foreign-trained, repatriated Pakistani physicians and examine its associations with affect balance and flourishing.

Methods

A cross-sectional survey was conducted between April 2022 and November 2023, recruiting 109 repatriated physicians via purposive sampling. Data was collected using the Scale of Positive and Negative Emotions (SPANE), the Flourishing Scale, and the SWL Scale. Relationships among these variables were assessed using correlation analysis, and logistic regression was used to identify predictors of SWL.

Results

Of the 109 participants, 70.6% were male, 83.5% resided in Punjab, 68.8% had trained in the US/Canada, and 69.7% worked in the private sector. The mean age was 47.31 (SD=7.9) years, and 58.7% held dual nationality. Overall, 90.8% reported being satisfied with life. Men (p=0.03) and married individuals (p=0.04) demonstrated higher SWL, whereas participants from Sindh and Khyber Pakhtunkhwa scored lower (p=0.007). SWL correlated moderately with flourishing (r=0.488), positive emotions (r=0.391), and affect balance (r=0.327). Each one-unit increase in flourishing was associated with a 1.236-fold higher odds of SWL (p=0.005).

Conclusions

Foreign-trained, repatriated Pakistani physicians in this study exhibited high SWL, closely linked to flourishing and positive affect. The repatriated physicians may significantly contribute to healthcare systems in lower-middle-income countries. Future research should examine broader samples, include locally trained or expatriated comparison groups, and explore repatriation challenges to further elucidate factors that influence physician well-being.

## Introduction

The migration of well-educated, qualified professionals and skilled personnel from low-resource, low-income, and developing countries to high-income countries, colloquially termed “brain drain,” has been ongoing for decades. In Pakistan, professionals such as engineers and healthcare providers, largely doctors and nurses, are at the forefront of the exodus. The United States, United Kingdom, Canada, and Australia are the leading recipients of international medical graduates (IMGs), mainly from lower-income countries [1,2].

IMGs add up to nearly one-third (23%-28%) of the total physician workforce in the United States, the United Kingdom, Australia, and Canada, and around half to two-thirds (40%-75.2%) of those are from low-income countries. Pakistan is one of the three leading source countries, following India and the Philippines, in supplying physicians to the aforementioned four developed countries. Besides, Pakistan has the second-highest “Emigration Factor” (11.7) in the Indian subcontinent, following Sri Lanka, indicating a higher number of Pakistani physicians working in the recipient countries and a lesser number in Pakistan, in comparison with other countries from the Indian Subcontinent [1].

As per 2002 statistics by the Bureau of Emigration and Foreign Employment, approximately 1000-1500 Pakistani doctors emigrate annually, with only 10%-15% returning to Pakistan later, thus contributing to a net migration of 900-1275 physicians. Moreover, the number of doctors that migrated from Pakistan to foreign countries between 2002 and 2011 was twice as high as that between 1971 and 2001 (11028 vs. 5085), showing the rising trend of Pakistani physicians leaving the country after graduation [3]. According to the Overseas Employment Corporation (OEC), more than 1600 doctors and 400 nurses proceeded to other countries for employment between 2007 and 2011, with the majority moving to the Middle East. However, there are no official records and data related to the exact number of returning migrant physicians who have repatriated to Pakistan after completing their postgraduate training abroad [4].

The self-reported driving factors of physician migration from across the developing world to developed countries also include economic and political instability, low-paid jobs, unfavorable workplace environments, and poor quality of postgraduate training programs in their home countries [5]. The reasons for Pakistani physicians’ emigration to foreign countries include professional development through better career opportunities due to long-standing beliefs about higher-quality, structured, and rigorous training programs; fair and merit-based service/career structures; and much higher remuneration in destination countries [6].

This “brain drain,” or emigration of healthcare workers, has far-reaching consequences for the economic and health development of the lower-income source countries, largely through the loss of human capital created as a result of high investment in subsidized public medical education leading to a low physician-to-patient ratio, thereby compounding health inequities in already underserved and impoverished populations [1].

The discourse around the concept of “brain drain” generally focuses on the adverse outcomes for the healthcare systems of third-world source countries. There are “moral value” judgments on physicians leaving their home countries with scarce healthcare workforces and high burdens of disease, and “moral hazards” for developed countries whose stable healthcare systems are very much dependent on physicians who migrated from countries with already collapsing healthcare systems [2]. The beneficial impact of the returning trained physicians on the healthcare system of their source countries is rarely taken into account in the brain drain debate [7]. The numerous benefits of foreign-trained physicians repatriating to their home countries include a much higher quality of clinical practice and medical education based on international standards, as well as internationally collaborated clinical and public health research [6].

Rough statistics show that around 300 of the total 1000 US-trained Pakistani physicians had repatriated to Pakistan by 2007 [6]. However, there is no official mechanism or database in Pakistan to keep records and data of returning migrant physicians who have repatriated to Pakistan after foreign training and work experience abroad [4]. 

There is an extremely limited body of research available on psychological well-being and satisfaction with life(SWL) in physicians, particularly from low- and middle-income countries, Pakistan being one of them. Similarly, there is a dearth of prior research done to elicit life satisfaction and its predictors among physicians repatriated to poorer countries after being trained in the rich and developed world. 

This cross-sectional study aims to assess SWL, flourishing, and psychological well-being as the indices of happiness in repatriated foreign-trained Pakistani physicians. It also aims to examine the associations between SWL, flourishing, and affect balance, and to identify predictors of SWL within this population.

This work was previously presented as an abstract poster at the 33rd European Congress of Psychiatry, held in Madrid, Spain, from April 5 to April 8, 2025, and at the World Psychiatric Association's 24^th^ World Congress of Psychiatry, held in Mexico City on November 17, 2024. 

## Materials and methods

Study design and sampling

This descriptive and analytical cross-sectional survey was conducted from April 2022 to November 2023. 

Inclusion and exclusion criteria

The physicians of Pakistani origin, at least 30 years of age, who were trained in a foreign country in any specialty, stayed there for at least two years, repatriated, and had been residing in any province of Pakistan for at least six months, were included. Those who repatriated to Pakistan less than six months before participation, were staying back only due to visa issues for the short term, and those who did not give consent were excluded.

Ethical considerations and consent

The Institutional Review Board (IRB) of King Edward Medical University (KEMU), Lahore, Pakistan, approved the study vide approval letter No. 417/ARA/KEMU dated 29/03/2022. Informed consent was sought from all the study participants, and the online questionnaires included the consent statement.

Data collection procedure

The data were collected through self-administered, online questionnaires using a non-probability purposive sampling technique (Appendix A). These questionnaires were distributed through the social media groups of the repatriated physicians, and reminders were sent via the groups or email addresses, if available. We also sent out the online questionnaire link to professors and departmental heads to get it filled in by any repatriated physicians working there. As there were no official records and data related to the exact number of returning migrant physicians who have repatriated to Pakistan after completing their postgraduate training or working abroad, we did not have a formal, defined sampling frame. The component on background and demographic characteristics comprised age, gender, marital status, number of children, nuclear or joint family, specialty, duration of stay in the foreign country, time since repatriation, public, private, or both types of practice in Pakistan, total professional experience, and dual nationality. Specialties included psychiatry, pediatrics, medicine and allied fields encompassing general medicine, emergency medicine, pulmonology, cardiology, nephrology, neurology, oncology, and gastroenterology, and surgery and allied fields encompassing general surgery, cardiothoracic surgery, neurosurgery, ophthalmology, otorhinolaryngology, gynecology and obstetrics, and other specialties (basic sciences, pathology, public health, physical therapy, and hospital administration).

Study instruments

The SWL Scale is a five-item self-report tool, constructed by Diener and colleagues in 1985 as a metric for assessment of the cognitive-judgment component of subjective well-being. The scale measures satisfaction with overall quality of life as an index of subjective well-being. The participants express their level of agreement with each statement on a seven-point Likert scale as follows: 1= strongly disagree, 2= disagree, 3= slightly disagree, 4= neither agree nor disagree, 5= agree, 6= slightly agree, 7= strongly agree [8]. The higher overall score indicates a higher level of SWL. The total score is interpreted as follows: 31-35 (extremely satisfied), 26-30 (satisfied), 21-25 (slightly satisfied), 20 (neutral), 15-19 (slightly dissatisfied), and 10-14 (dissatisfied).

The eight-item Flourishing Scale is a self-report tool devised by Diener and others in 2009 that measures the respondents’ perceptions of success in different areas of life, including self-esteem, purpose, optimism, relationships, and professional competence, as a metric of psychological resources, well-being, and social prosperity [9]. It uses a seven-point Likert scale ranging from 1=strongly disagree to 7=strongly agree. The total flourishing score is calculated by adding the responses for all eight items. The scores range from eight to 56, indicating the lowest and the highest levels of psychological well-being, respectively [10].

The Scale of Positive and Negative Experiences (SPANE) is a 12-item questionnaire that measures the positive and negative feelings experienced by the respondents over the past month on the five-point Likert scale: 1=very rarely or never, 2=rarely, 3=sometimes, 4=often, and 5=very often or always. Its six items are specific for positive emotions (Positive Emotions Subscale (SPANE-P)), and the other six for negative emotions (Negative Emotions Subscale (SPANE-N)). The two subscales have three general items each, such as pleasant, good, and positive for SPANE-P. Both the subscales are scored ranging from 1 to 5 each, and the overall affect balance is calculated by subtracting the SPANE-N scores from the SPANE-P scores, which may range from -24 (the lowest possible affect balance/unhappiest possible) to 24 (the highest possible affect balance/the happiest possible). 

Data analysis 

Data were analyzed using IBM SPSS Statistics for Windows, version 25.0 (IBM Corp., Armonk, NY, USA). The descriptive statistical results for background and demographic characteristics were demonstrated as frequencies (f) and percentages (%).

Normality analysis using histograms, QQ plots, Kolmogorov-Smirnov, and Shapiro-Wilk tests showed that our data were not normally distributed for the outcome variable of SWL. Hence, we used the Inverse Distribution Fraction (IDF) transformation method to normalize the data and apply parametric tests for inferential statistical analysis. We also used IDF transformation for Flourishing, SPANE-P, and SPANE-N Scales [11].

The outcome variable (SWL) was computed as a dichotomous variable with the following cutoff scores: 5-20 (dissatisfaction with life) and 21-35 (SWL).

The continuous SWL scores were dichotomized into ‘satisfaction’ and ‘dissatisfaction’ categories using a cutoff of 20 reported in previously published literature [12]. This decision to dichotomize was made primarily to facilitate regression analysis while minimizing the impact of missing item data in individual cells, which could have been best ensured using the binary logistic regression analysis.

We applied t-tests and one-way analysis of variance (ANOVA) for comparison of total mean scores of the Satisfaction with Life scale across subcategories of demographic and background characteristics. The value for 'α’ was set at 0.05. 

Pearson's correlation test was used to analyze the relationship between SWL, flourishing, and overall affect balance. The Pearson's correlation coefficient (r) values of +1 and -1 indicate a perfect linear positive and negative correlation, respectively, and 0 indicates none. +0.7-+0.9 and -0.7- -0.9 represent strong positive and inverse correlations, respectively. Similarly, 0.4-0.6 indicates a moderate and 0.1-0.3 indicates a weak positive correlation, -0.4 - -0.6 indicates a moderate negative, and -0.3 - -0.1 shows a weak negative relationship [13].

The binary logistic regression analysis was employed to determine the predictor factors for SWL with satisfaction or dissatisfaction with life as the outcome and flourishing and overall affect balance as the predictor variables. We tested assumptions of no multicollinearity through collinearity diagnostics of the Variance Inflation Factor (VIF) and Condition Index. The tests of model fitness, Omnibus Tests of Model Coefficients, and Hosmer and Lemeshow tests were significant at α < 0.05 and > 0.05, respectively. Odds ratios (ORs) were calculated for SWL at α=0.05.

## Results

Descriptive results

One hundred and nine participants responded to the questionnaire. The average age of study respondents was 47.31 ± 7.97 years (SD). The majority (70.6%) of the participants were males, currently married (89.9%), from the province of Punjab (83.4%), and in the age group 41-50 years (45%). Around two-thirds (69.7%) were working in the private sector only. 68.8% were trained in the USA or Canada, and 25.7% in the UK, Ireland, or Australia. A majority (40.3%) had repatriated to Pakistan recently, one to five years back (Table 1).

**Table 1:** Demographic and background characteristics of the study participants SD: Standard Deviation; n (%): Number (Percentage); KPK: Khyber Pakhtunkhwa; ICT: Islamabad Capital Territory; AJK: Azad Jammu and Kashmir; FATA: Federally Administered Tribal Areas; GB: Gilgit-Baltistan; KSA: Kingdom of Saudi Arabia; UAE: United Arab Emirates; SWL: Satisfaction with Life; USA: United States of America; UK: United Kingdom

Characteristic	Category	n (%)	Mean ± SD
Age (years)	Continuous		47.31 ± 7.97
	30–40	21 (19.3%)	
	41–50	49 (45.0%)	
	51–60	34 (31.1%)	
	>60	5 (4.6%)	
Gender	Male	77 (70.6%)	
	Female	32 (29.4%)	
Marital status	Never married/Divorced/Widowed	11 (10.1%)	
	Married	98 (89.9%)	
Number of children	None	6 (5.6%)	
	≤2	43 (39.4%)	
	≥3	60 (55.0%)	
Family system	Nuclear	63 (57.8%)	
	Joint	46 (42.2%)	
Province	Punjab	91 (83.4%)	
	Sindh	9 (8.3%)	
	KPK	6 (5.5%)	
	ICT/AJK/FATA/GB	3 (2.8%)	
Training location	USA/Canada	75 (68.8%)	
	UK/Ireland/Australia	28 (25.7%)	
	KSA/UAE	2 (1.8%)	
	Other	4 (3.7%)	
Time stayed abroad	<5 years	23 (21.1%)	
	5–10 years	20 (18.3%)	
	11–15 years	31 (28.3%)	
	16–20 years	22 (20.2%)	
	≥21 years	13 (11.9%)	
Time since repatriation	<1 year	11 (10.1%)	
	1–5 years	44 (40.3%)	
	6–10 years	21 (19.3%)	
	11–15 years	15 (13.8%)	
	16–20 years	10 (9.2%)	
	≥21 years	8 (7.3%)	
Specialty	Medicine & Allied Sciences	57 (52.3%)	
	Surgery & Allied Sciences	20 (18.3%)	
	Psychiatry	19 (17.4%)	
	Pediatrics	7 (6.4%)	
	Other	6 (5.5%)	
Experience	<5 years	12 (11%)	
	5–10 years	18 (16.5%)	
	11–15 years	19 (17.4%)	
	16–20 years	29 (26.6%)	
	≥21 years	31 (28.3%)	
Work setting	Public	18 (16.5%)	
	Private	76 (69.7%)	
	Both	15 (13.8%)	
Dual nationality	Yes	64 (58.7%)	
	No	45 (41.3%)	
SWL category	Satisfied	99 (90.8%)	
SWL category	Dissatisfied	10 (9.2%)	

The mean score for total life satisfaction was 27.48 ± 5.03 SD. 90.8% (n=99) were satisfied with life, while 10 (n=9.2%) were dissatisfied with life. Most of the study respondents (45%, n=49) chose being satisfied with life. 25.7% (n=28) were extremely satisfied, and 22 (20.2%) participants were slightly satisfied. 1.8% were neutral, 1.8% were dissatisfied, and 5.5% were slightly dissatisfied. 

The Flourishing Scale, another indicator of well-being, had a mean score of 47.89 ± 6.79. The SPANE-P subscale yielded a mean score of 24.60 ± 3.72 SD. Similarly, the SPANE-N subscale yielded a mean of 13.58 ± 3.85 S.D. 

SWL scores across background and demographic characteristics

As displayed in Table 2, we found statistically significant differences in the total SWL scale scores across subcategories of gender (p=.03), marital status (p=.04), and provinces (p=.007). We found that the divorced/separated/widowed/never-married participants had comparatively lower scores on the SWL scale (24.63 ± 5.49) than currently married participants (27.80 ± 4.90) at p = .04. We also found that participants from different provinces had differences in Satisfaction with Life scores at p = .007, with the highest scores reported from Islamabad, Azad Jammu and Kashmir, Federally Administered Tribal Areas, and Gilgit-Baltistan, followed by Punjab. We did not find any statistically significant differences in life satisfaction scores across genders, specialties, age groups, public or private sector, total clinical experience, nuclear or joint family system, and time duration since repatriation. 

**Table 2:** SWL scores across background characteristics SWL = Satisfaction with Life; SD = Standard Deviation; CI = Confidence Interval; KPK = Khyber Pakhtunkhwa; ICT = Islamabad Capital Territory; AJK = Azad Jammu and Kashmir; FATA = Federally Administered Tribal Areas; GB = Gilgit-Baltistan; KSA = Kingdom of Saudi Arabia; UAE = United Arab Emirates.

Characteristic	Category	Mean ± SD	95% CI	p-value
Total SWL Score		27.48 ± 5.03		
1. In most ways, my life is close to my ideal.		5.28 ± 1.297		
2. The conditions of my life are excellent.		5.48 ± 1.244		
3. I am satisfied with my life.		5.69 ± 1.317		
4. So far I have gotten the important things I want in life.		5.90 ± 1.154		
5. If I could live my life over, I would change almost nothing.		5.06 ± 1.699		
Age in categories	30 - 40 years	28.67 ± 4.60	(26.57 - 30.77)	.30
Age in categories	41 - 50 years	26.50 ± 5.63	(24.88 - 28.12)	
Age in categories	51 - 60 years	28.14 ± 4.45	(26.59 - 29.70)	
Age in categories	More than 60 years	27.53 ± 3.17	(23.59 - 31.46)	
Gender	Male	27.69 ± 4.61	(26.65 – 28.74)	.03
Gender	Female	26.95 ± 5.97	(24.80 – 29.11)	
Marital status	Never married / Divorced / Separated / Widowed	24.63 ± 5.49	(20.94 - 28.32)	.04*
Marital status	Currently married	27.80 ± 4.90	(26.81 - 28.78)	
Number of children	None	25.85 ± 6.78	(18.73 – 32.97)	.72
Number of children	<=2	27.60 ± 5.24	(25.99 – 29.22)	
Number of children	3 or above	27.55 ± 4.75	(26.32 – 28.78)	
Family system	Nuclear family	27.27 ± 5.14	(25.98 - 28.57)	.62
Family system	Joint family	27.75± 4.91	(26.29 - 29.21)	
Province	Punjab	27.52 ± 4.75	(26.53 – 28.51)	.007*
Province	Sindh	25.58 ± 5.96	(20.99 - 30.17)	
Province	KPK	25.87 ± 4.86	(20.17 - 30.38)	
Province	ICT, AJK, FATA, GB	36.28 ± 1.19	(33.21 - 39.25)	
Foreign country of training	USA, Canada	27.64 ± 4.81	(26.53 - 28.75)	.93
Foreign country of training	UK, Ireland, Australia	27.26 ± 4.99	(25.32 - 29.19)	
Foreign country of training	KSA, UAE	25.87 ± 9.55	(59.95 - 111.70)	
Foreign country of training	Other	27.48 ± 5.03	(12.41 - 41.08)	
Duration of stay in foreign country	<5 years	26.78 ± 5.61	(24.35 - 29.21)	.75
Duration of stay in foreign country	5 - 10 years	28.05 + 4.00	(26.18 - 29.92)	
Duration of stay in foreign country	11 - 15 years	27.84 ± 5.89	(25.67 - 30.00)	
Duration of stay in foreign country	16 - 20 years	26.63 ± 4.05	(24.83 - 28.42)	
Duration of stay in foreign country	21 years or more	28.41 ± 4.98	(25.39 - 31.42)	
Duration since repatriation	<1 year	27.51 ± 5.89	(23.55 - 31.47)	.92
Duration since repatriation	1 - 5 years	27.21 ± 5.17	(25.64 - 28.78)	
Duration since repatriation	6 - 10 years	26.84 ± 4.16	(24.94 - 28.73)	
Duration since repatriation	11 - 15 years	28.44 ± 5.93	(25.15 - 31.72)	
Duration since repatriation	16 - 20 years	27.55 ± 5.07	(23.93 - 31.18)	
Duration since repatriation	21 years or more	28.70 ± 4.33	(25.07 - 32.32)	
Specialty	Medicine and Allied	28.22 ± 4.89	(26.92 - 29.52)	.10
Specialty	Surgery and Allied	25.08 ± 4.28	(23.08 - 27.08)	
Specialty	Psychiatry	28.26 ± 6.03	(25.35 - 31.17)	
Specialty	Pediatrics	26.28 ± 3.65	(22.89 - 29.66)	
Specialty	Others	25.06 ± 3.04	(20.21 - 29.90)	
Duration of work in specialty	<5 years	28.78 ± 4.52	(25.91 - 31.66)	.71
Duration of work in specialty	5 - 10 years	26.24 ± 5.76	(23.38 - 29.11)	
Duration of work in specialty	11 - 15 years	27.85 ± 5.29	(25.30 - 30.41)	
Duration of work in specialty	16 - 20 years	27.19 ± 5.47	(25.10 - 29.27)	
Duration of work in specialty	21 years or more	27.73 ± 4.27	(26.16 - 29.29)	
Current employment/work	Public sector	26.19 ± 3.22	(24.59 - 27.80)	.24
Current employment/work	Private sector	28.01 ± 5.05	(26.86 - 29.17)	
Current employment/work	Both	26.30 ± 6.39	(22.69 - 29.84)	
Dual Nationality	Yes	27.41 ± 5.05	(26.15 - 28.67)	.87
Dual Nationality	No	27.57 ± 5.06	(26.05 - 29.09)	

Correlation between SWL, flourishing, positive and negative experiences, and overall affect balance

Table 3 shows that the strongest positive correlation was found between positive emotions (SPANE-P scores) and Total Affect Balance (r=0.851), followed by a moderate positive correlation (r=0.648) between flourishing and positive emotions (SPANE-P). Further, our results showed moderate positive correlations between SWL and flourishing (r=0.488) and SWL and total affect balance (r=0.413). We found a strong negative correlation between total affect balance and negative emotions (SPANE-N), a moderate correlation between SPANE-P and SPANE-N scores, and a weak negative relationship of negative emotions (SPANE-N) with SWL (-.327) and flourishing (-.396). All correlations were significant at p < 0.001. 

**Table 3:** Correlation between satisfaction with life, flourishing, positive and negative emotions, and overall affect balance **. Correlation is significant at the 0.01 level (2-tailed) SWL: Satisfaction with Life Scale; SPANE-P: Scale of Positive and Negative Experience – Positive Affect; SPANE-N: Scale of Positive and Negative Experience – Negative Affect.

	SWL	Flourishing	SPANE-P	SPANE-N	Overall Affect balance
SWL	1				
						Flourishing	0.488^**^	1			
SPANE P	0.391^**^	0.648^**^	1		
SPANE N	-.327^**^	-.396^**^	-.471^**^	1	
Overall Affect Balance	0.413^**^	0.584^**^	0.851^**^	-.846^**^	1

Binary logistic regression

The assumptions of a binary/dichotomous response variable with independent and mutually exclusive subcategories were met. We found a high variance inflation factor for SPANE-P (15.046), SPANE-N (14.324), and overall affect balance (39.502), indicating strong collinearity. The Condition Index was found to be high (67.522) in one dimension with a high variance proportion for SPANE-P (0.98), SPANE-N (0.83), and overall affect balance (0.94). Therefore, SPANE-P and SPANE-N were removed from the final model due to high multicollinearity with the overall affect balance. 

The final model with SWL as the outcome variable and flourishing and overall affect balance as predictor variables was statistically significant (X2 (2, N=109, p=0.001)). Omnibus tests of model coefficients (p = .001) and Hosmer and Lemeshow tests (p = .322) showed good model fitness with the data. The percentage accuracy in classification (PAC) was 90.8%, which showed that our model predicted the correct category well enough when the predictors were added, correctly classifying 90.8% of cases with a sensitivity of 99%. Cox and Snell R² and Nagelkerke R² showed that 12.6-27.5% variability in the response variable (satisfaction with life) was due to predictor variables. 

Our results showed that each one-unit increase in flourishing led to 1.236 times higher odds of SWL (p = .005); hence, flourishing was a predictor of SWL. On the other hand, the overall affect balance was not found to be a statistically significant predictor of SWL (Table 4).

**Table 4:** Binary logistic regression analysis results showing predictors of satisfaction with life. B: Unstandardized Regression Coefficient; SE: Standard Error of the Coefficient; OR: Odds Ratio; CI: Confidence Interval; p: Probability Value.

Predictor Variable	B	S.E	OR	95% CI for OR		p
				Lower Bound	Upper Bound	
Flourishing	0.213	0.076	1.236	1.066	1.437	.005*
Overall Affect Balance	0.024	0.058	1.025	0.914	1.148	.677

## Discussion

The present study explored SWL and its contributing factors among Pakistani physicians who repatriated after their postgraduate training in a foreign country. The results indicated that flourishing, affect balance, gender, marital status, and the province played a significant role in physicians’ overall satisfaction with their lives after repatriation to Pakistan. Given the stressful and demanding nature of the medical profession, these findings could play an important role in understanding a physician’s mental health and provide clues to how we can better support the mental health of repatriated physicians [14].

Psychological well-being connotes experiencing positive emotions, functioning well in personal life and effectively at work, engaging in and experiencing positive relationships, developing one’s potential, and being confident, happy, and content with life [15]. Individual well-being is considered the direct antecedent of SWL and an indirect predictor of overall happiness [16]. Prior research shows that satisfaction with different domains or areas of life, such as financial situation, family and social support, health, job and work, and self-worth in workers, predicts overall SWL [17]. A study from Australia showed that social policy, human rights, and socioeconomic situations in a country also predict happiness and SWL in people [18].

Prior research has also demonstrated that predictors of life satisfaction tend to be culture-specific and vary across the economic rankings and the individualist or collectivist culture of a country. According to the World Value Survey findings, satisfaction with esteem needs (confidence, self-worth, social capital, and respect) tends to be the major predictor of SWL in individualist compared to collectivist nations. Likewise, financial satisfaction is more strongly correlated with life satisfaction in lower-income or poorer countries and personal or home life satisfaction in wealthier countries [19]. A Norwegian nationwide longitudinal study lasting 10 years showed that physicians were generally less satisfied with life than the general population, with younger age, married marital status, high perceived social support, and personality dynamics as the major predictors of higher levels of SWL [20]. 

Flourishing is a widespread concept that covers a sense of accomplishment across various dimensions, such as academic achievements, relationships, goals and purpose in life, professional competence, and prospects. Flourishing positively correlated with life satisfaction, suggesting that physicians who perceived that they had succeeded in important areas of their lives were happy and content. Ryff and Singer (2008) reported that individuals with a strong sense of purpose and meaning experienced higher levels of life satisfaction [21]. These findings are consistent with previous research, which has established flourishing as an important aspect of psychological well-being [22]. One plausible explanation of flourishing as a predictor of life satisfaction in our participants is that the foreign-trained physicians had higher self-confidence and self-efficacy levels in terms of their medical knowledge and skills, thus reflecting their sense of flourishing and satisfaction in life. 

Physicians with better emotional regulation and an optimal balance between positive and negative emotions were found to be more satisfied with their lives. While physicians are generally expected to have better emotional control and regulation, what Sir William Osler (1904) referred to as ‘imperturbability,’ medical training does not necessarily teach or train future physicians in this important soft skill [23, 24]. In our study, the experience of positive emotions was related to higher levels of life satisfaction, while the experience of negative emotions was related to lower levels of life satisfaction. These findings were consistent with prior research, which concluded that emotions play a key role in life satisfaction [25]. The experiences of positive emotions such as happiness, joy, enthusiasm, and optimism are likely to result in increased self-efficacy, which in turn can lead to better adjustment and satisfaction with career-related initiatives after repatriation. Nevertheless, experiences of negative emotions such as distress, frustration, and pessimism stemming from structural and cultural differences in the workplace may result in decreased career-related satisfaction. 

It is also interesting to note that married physicians had comparatively higher levels of life satisfaction. These marital disparities offer valuable insights into the structural and functional societal dynamics, particularly in a developing country like Pakistan. Research has indicated that men in traditional societies derive life satisfaction from professional achievements, while women’s overall life satisfaction may be affected by social judgments, feminine gender roles, expectations, and additional domestic and motherhood-related responsibilities [26, 27]. Studies also indicate that a robust social support network contributes to psychological well-being [28]. Being married provides such support in the form of love, care, and emotional strength from a spouse and children, helping in coping with work-related stressors and thus increasing SWL. 

A limitation of this study is its cross-sectional nature, which limits causal inferences and attributions as to whether life satisfaction is a consequence of flourishing or affect balance or its outcome. In order to determine the direction of these relationships, longitudinal and qualitative exploration of psychological well-being and satisfaction in life would be necessary aspects of future research in this domain. Moreover, these findings are specific only to repatriated physicians and do not allow comparisons between life satisfaction levels in the physicians who never migrated and those who are currently working abroad. We acknowledge that the heavy geographic concentration in Punjab may limit the national representativeness of these findings and that the overrepresentation of US/Canada-trained physicians may bias well-being outcomes upward. Additionally, the use of self-administered questionnaires has an associated risk of social desirability bias and recall bias, along with subjective interpretation.

Nonetheless, our study contributes meaningfully as a pioneer study, initiating a discourse on this emerging area of scientific inquiry and highlighting the relationship between SWL, flourishing, and affect balance.

We recommend qualitative exploration of the lived experiences of repatriated physicians, the reasons for their repatriation, the challenges they faced after repatriation, and factors contributing to their life satisfaction, including relevant predictors, i.e., income, workload, job security, institutional support, discrimination, or reintegration challenges, to be the focus of research on this subject. This project can be further extended to those physicians who stay on in foreign countries after completing their training to find the reasons and motivations behind these decisions and the challenges they face. Future studies can also add to the cultural adaptation of the quantitative instruments in the specific context of repatriated Pakistani physicians.

Further exploration to fill in research gaps in this emerging area, and the findings have implications not only for the better well-being of repatriated physicians but also for improved service delivery through the integration of highly skilled and well-trained repatriated physicians in local infrastructure. Competitive salaries, social recognition of repatriated physicians’ contribution to the health sector, reduced political control and bureaucratic hurdles, improved autonomy and agency through involvement in policy decisions, merit-based career advancement opportunities, and better retention strategies could be managerial and policy-level interventions to improve SWL in foreign-trained physicians. This can help to reintegrate repatriated physicians back into Pakistan’s severely depleted healthcare systems, as well as devise strategies and policies to overcome the perpetual problem of medical “brain drain,” one of the biggest challenges in healthcare system development in a “third world” country such as Pakistan.

## Conclusions

Repatriated, foreign-trained Pakistani physicians in this sample reported high SWL. Life satisfaction scores showed a moderate positive correlation with flourishing and positive affect. Flourishing also emerged as a significant predictor of life satisfaction. Future research should include larger, comparative samples as well as qualitative exploration of the lived experiences of repatriated physicians to better understand the factors that support or challenge the well-being of physicians after returning home. Additionally, future work employing a longitudinal approach would help clarify temporal relationships and provide deeper insight into the reintegration experiences of these physicians.

## Figures and Tables

**Figure 1:**
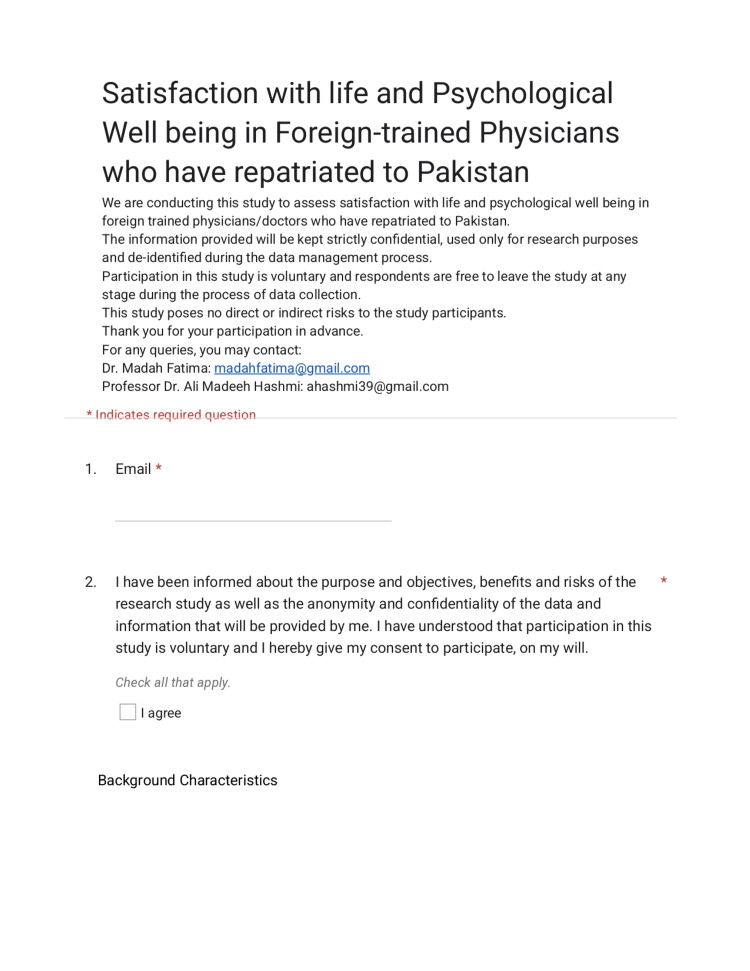
Page 1 of the questionnaire

**Figure 2:**
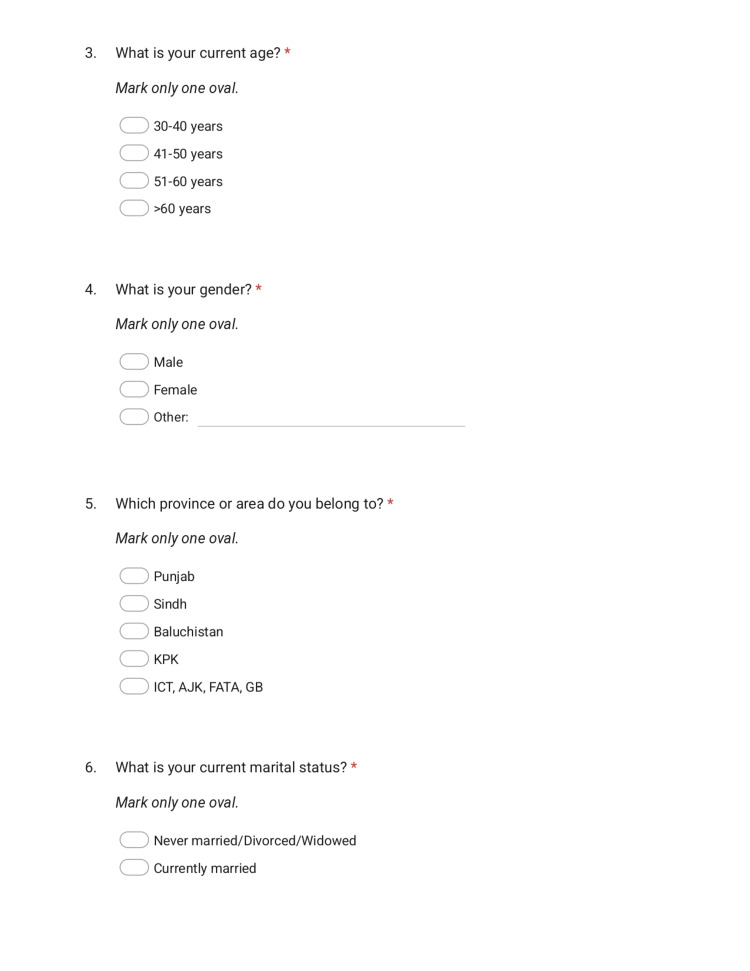
Page 2 of the questionnaire

**Figure 3:**
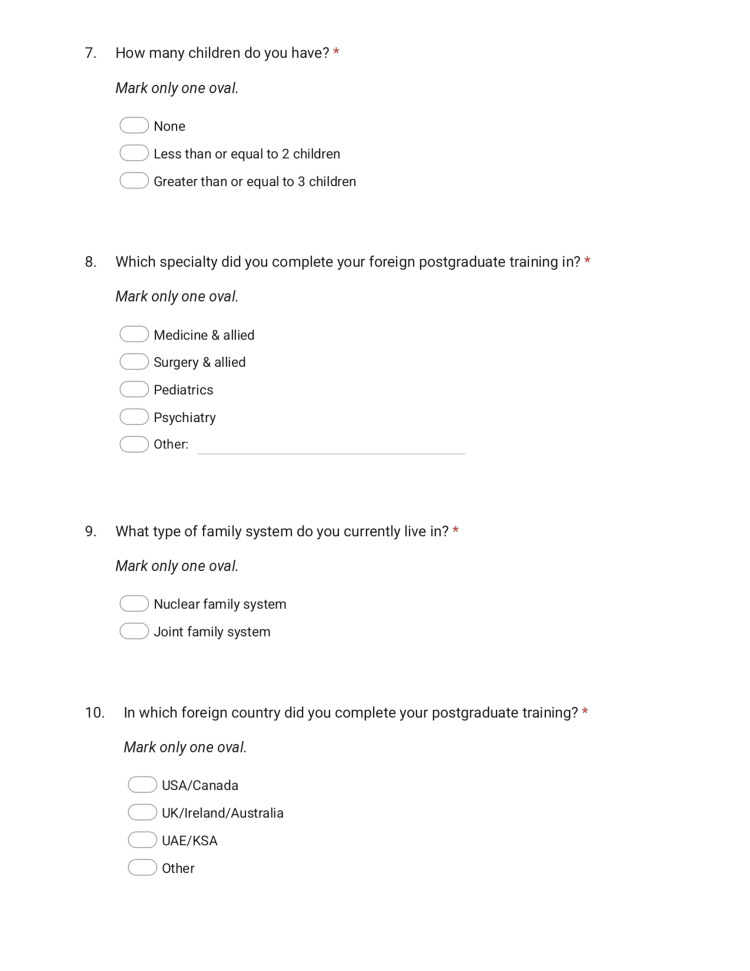
Page 3 of the questionnaire

**Figure 4:**
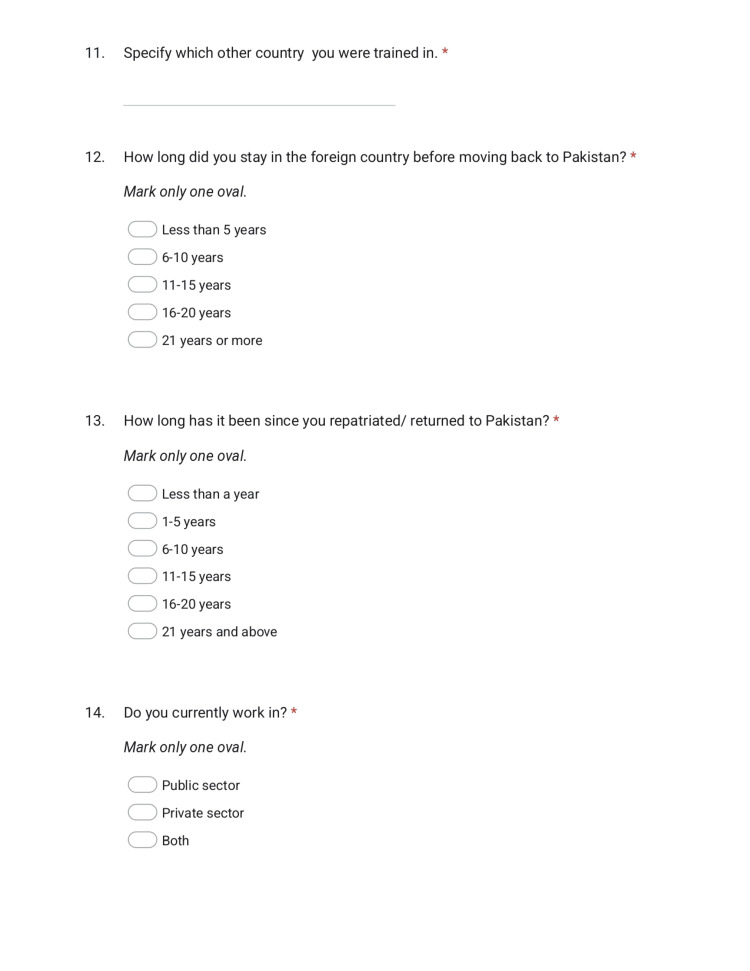
Page 4 of the questionnaire

**Figure 5:**
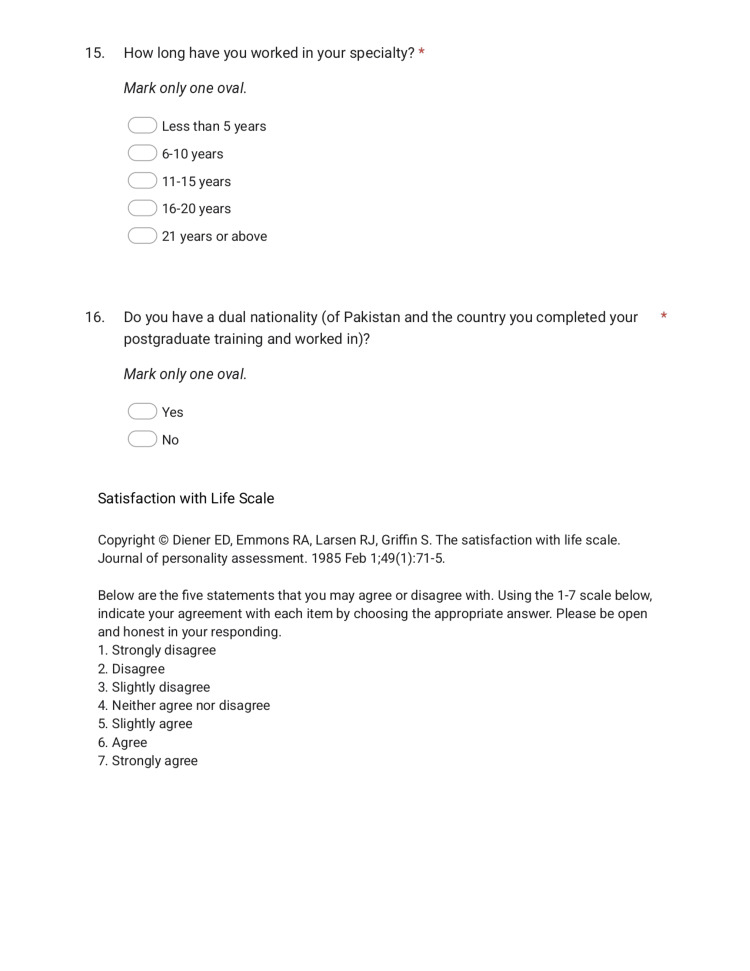
Page 5 of the questionnaire

**Figure 6:**
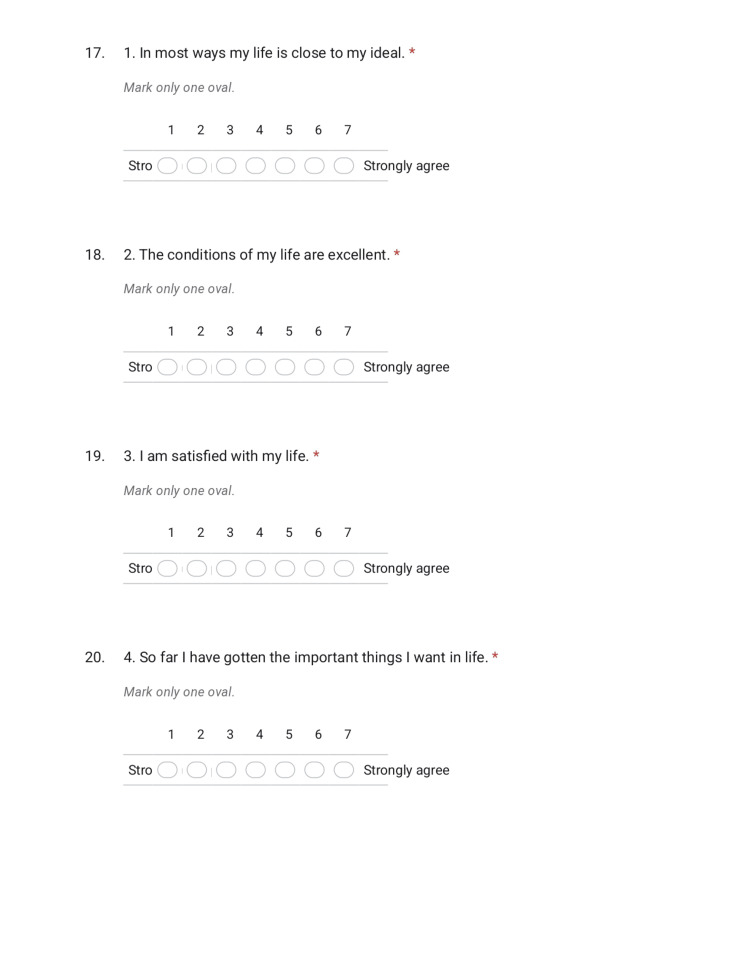
Page 6 of the questionnaire

**Figure 7:**
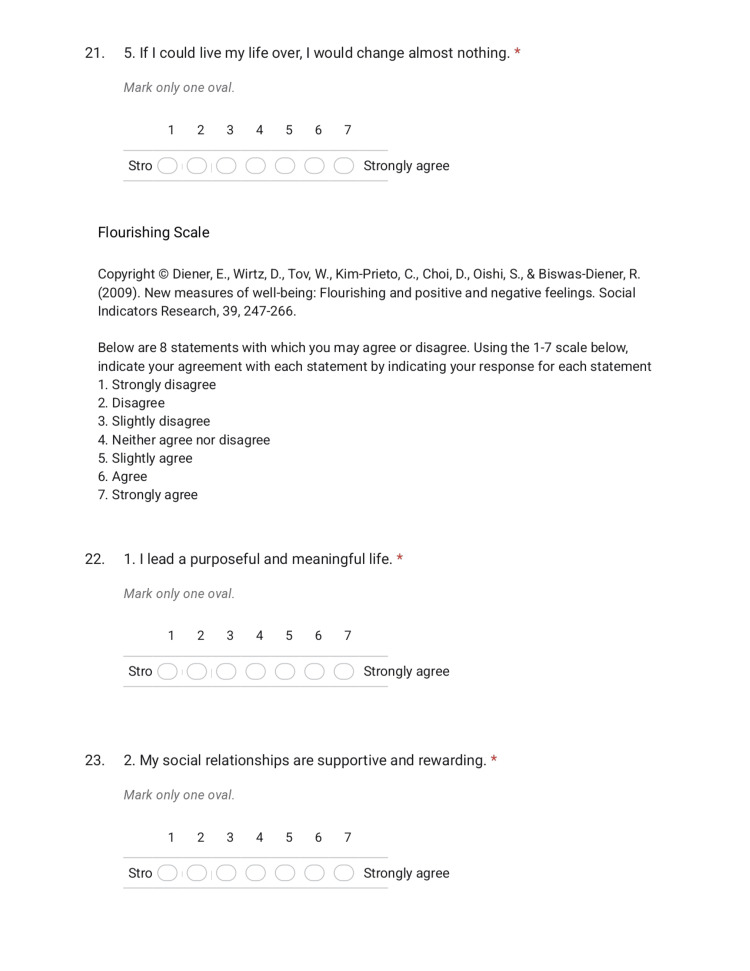
Page 7 of the questionnaire

**Figure 8:**
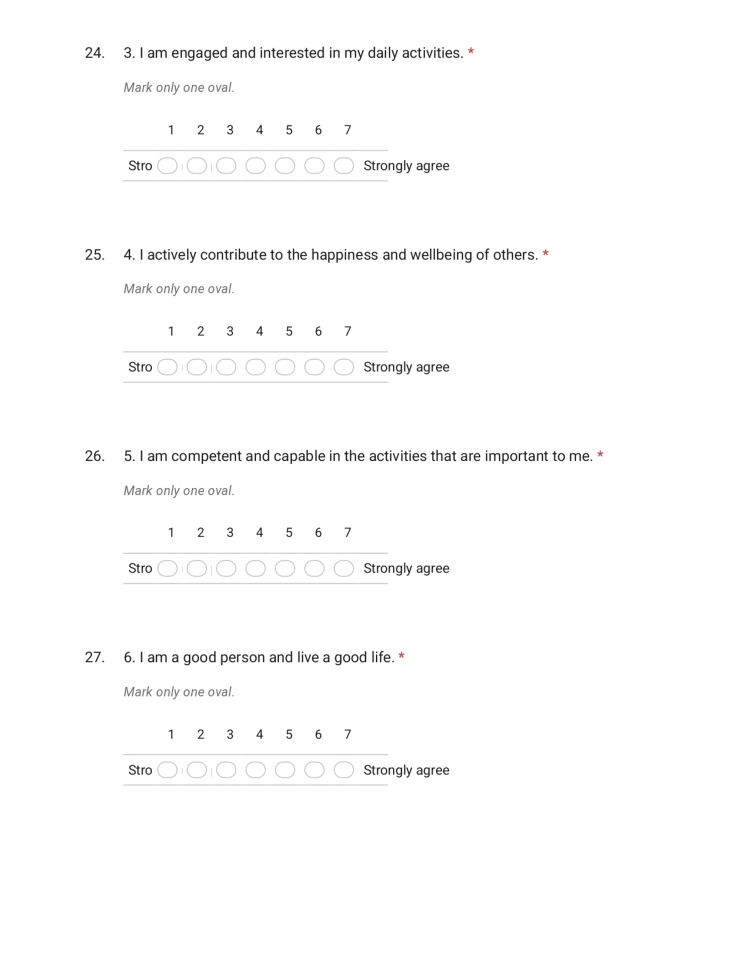
Page 8 of the questionnaire

**Figure 9:**
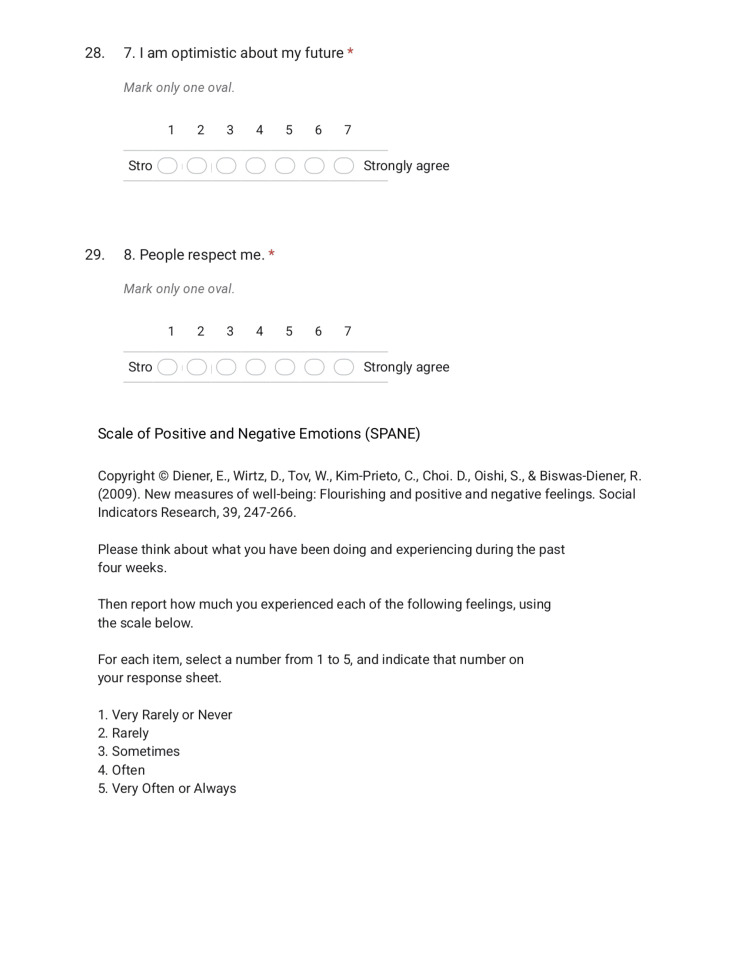
Page 9 of the questionnaire

**Figure 10:**
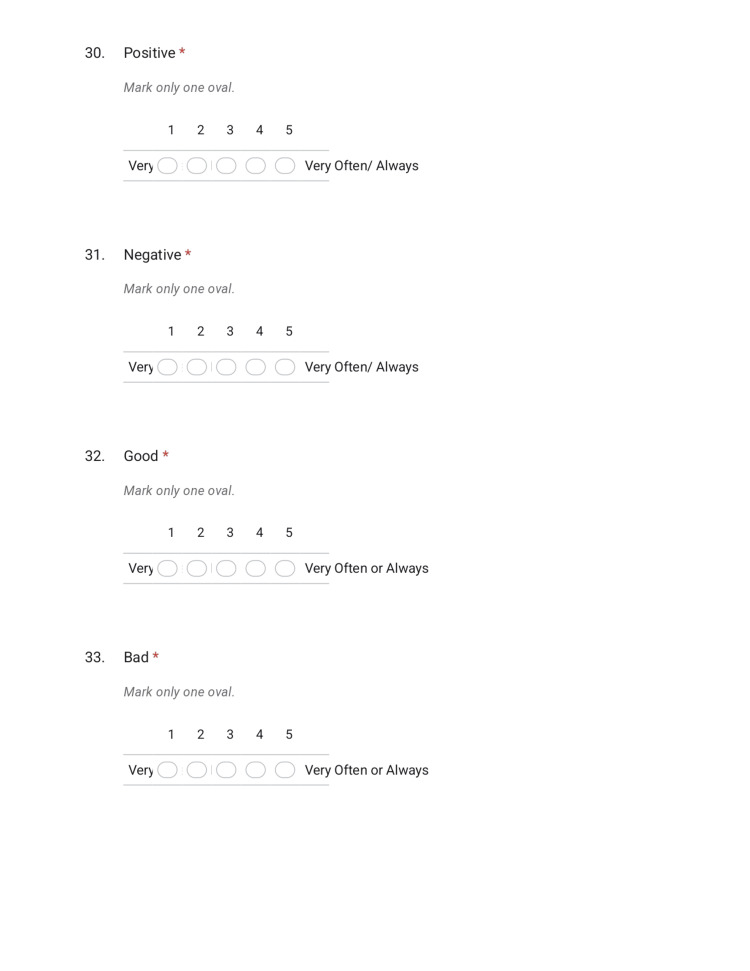
Page 10 of the questionnaire

**Figure 11:**
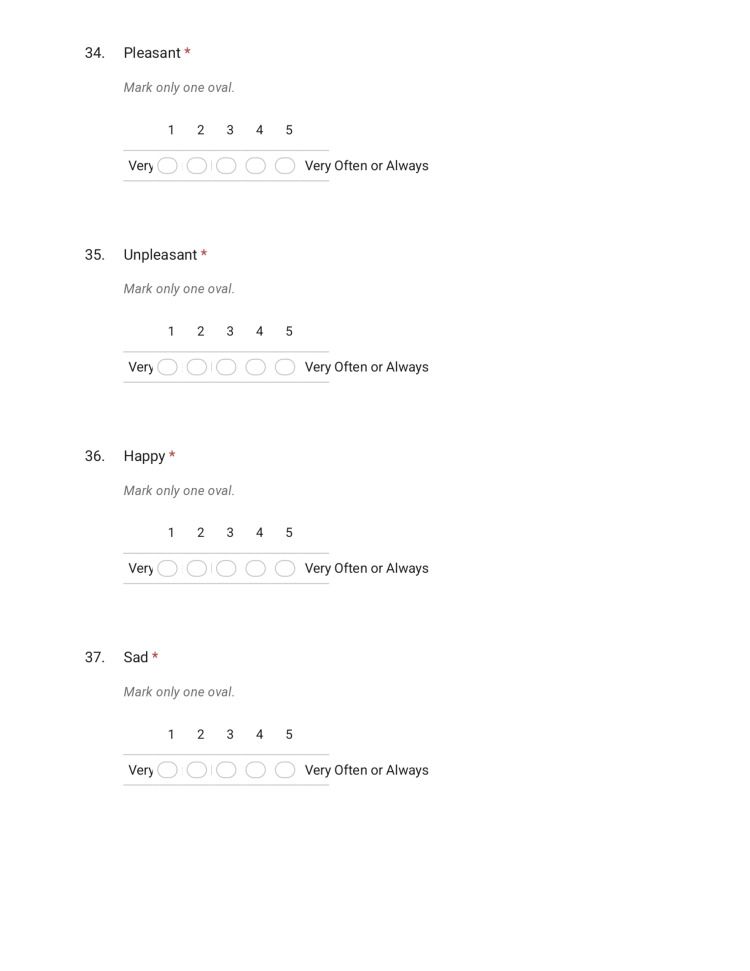
Page 11 of the questionnaire

**Figure 12:**
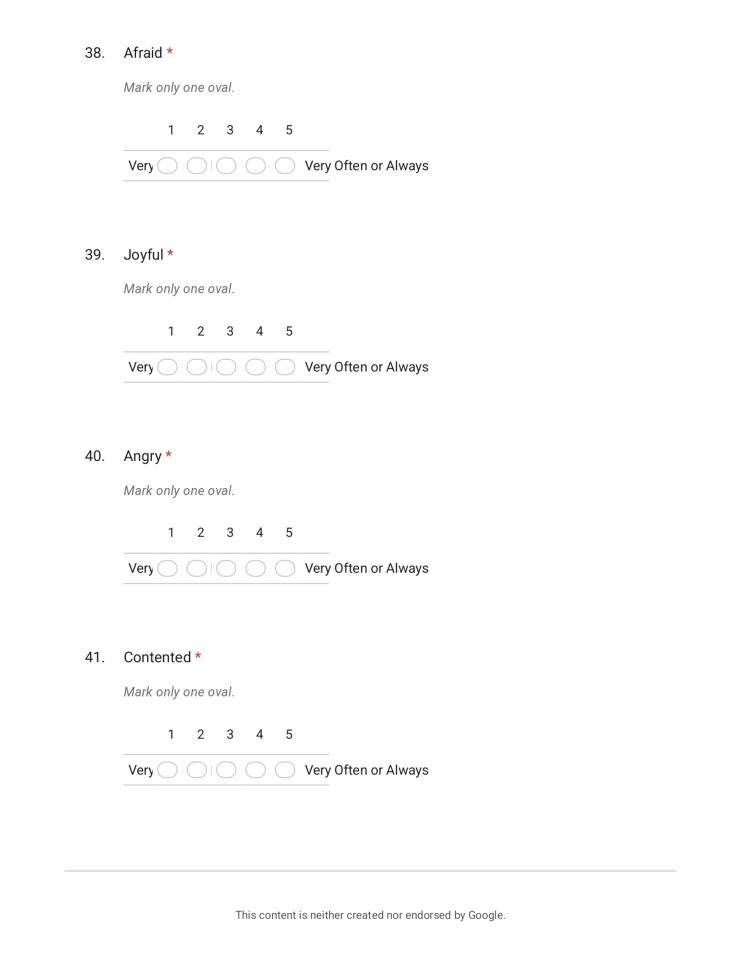
Page 12 of the questionnaire

## Appendices

Appendix A
